# A Patient-Specific Computational Framework for the Argus II Implant

**DOI:** 10.1109/OJEMB.2020.3001563

**Published:** 2020-06-11

**Authors:** Kathleen E. Finn, Hans J. Zander, Robert D. Graham, Scott F. Lempka, James D. Weiland

**Affiliations:** Department of Biomedical Engineering, University of Michigan, Ann Arbor, MI, USA and are associated with the Biointerfaces Institute.; Department of Biomedical Engineering, University of Michigan, Ann Arbor, MI, USA and are associated with the Biointerfaces Institute.; Department of Biomedical Engineering, University of Michigan, Ann Arbor, MI, USA and are associated with the Biointerfaces Institute.; Department of Biomedical Engineering, University of Michigan, Ann Arbor, MI, USA and are associated with the Biointerfaces Institute.; Department of Biomedical Engineering, University of Michigan, Ann Arbor, MI, USA and are associated with the Biointerfaces Institute.

**Keywords:** retinal prosthesis, Argus II, computational modeling, retinal ganglion cell, patient-specific

## Abstract

**Goal:**

Retinal prosthesis performance is limited by the variability of elicited phosphenes. The stimulating electrode’s position with respect to retinal ganglion cells (RGCs) affects both perceptual threshold and phosphene shape. We created a modeling framework incorporating patient-specific anatomy and electrode location to investigate RGC activation and predict inter-electrode differences for one Argus II user.

**Methods:**

We used ocular imaging to build a three-dimensional finite element model characterizing retinal morphology and implant placement. To predict the neural response to stimulation, we coupled electric fields with multi-compartment cable models of RGCs. We evaluated our model predictions by comparing them to patient-reported perceptual threshold measurements.

**Results:**

Our model was validated by the ability to replicate clinical impedance and threshold values, along with known neurophysiological trends. Inter-electrode threshold differences *in silico* correlated with *in vivo* results.

**Conclusions:**

We developed a patient-specific retinal stimulation framework to quantitatively predict RGC activation and better explain phosphene variations.

## I. INTRODUCTION

RETINITIS pigmentosa (RP) is a progressive degenerative disease that causes severe blindness, affecting over a million people worldwide [1]. The disease results in photoreceptor death, preventing the transduction of light into neural signals. However, even in end stages of RP, 30% of RGCs and 60% of bipolar cells remain intact [2]. Retinal prostheses use electrodes to activate these remaining retinal cells and evoke visual percepts [3]. One such system, the Argus II, has been implanted in over 350 patients worldwide. This system induces phosphenes (“spots of light”) for profoundly blind subjects, enabling improvements in mobility, orientation, and vision-related quality of life [4], [5]. However, these functional outcomes vary substantially among patients and perceptual resolution with the implant is limited. While users gain light sensitivity, they typically remain in the ultra-low vision range, below the level of standard visual acuity tests [4].

Although phosphenes are consistent for a single electrode from trial to trial, they vary across subjects and electrodes [6], [7]. Understanding phosphene variability is essential for improving retinal stimulation strategies and generating useful prosthetic vision. Electrode-retina distance has been shown to affect the charge threshold required to induce visual perception [8], [9]. The heterogeneity of retinal degeneration also impacts perceptual thresholds by altering retinal thickness and the number of viable RGCs [10], [11]. Electrode position in relation to ganglion axon pathways affects phosphene shape, due to activation of passing axon fibers [7]. Over half of Argus II patients have a foreign body response causing fibrotic tissue growth around the microelectrode array (MEA) post-implantation, but effects on perception remain unknown [12]. Finally, the position of the extraocular current return in relation to stimulating electrodes will shape the electric field and may influence RGC activation.

We hypothesize that a patient-specific computational framework can capture the aforementioned factors to model and explain the neurophysiological mechanisms causing phosphene variability. Existing finite element models (FEMs) of retinal stimulation have simplified the retina as a slab of homogenous tissue with electrodes positioned at a uniform distance from the retina [11], [13]–[17]. These models are unable to predict a different retinal response between electrodes, and therefore cannot explain phosphene variability. Furthermore, incorporating imaging data to create patient-specific models has proven beneficial for optimizing stimulation parameters for other neuromodulation therapies, such as deep brain stimulation [18], [19].

Here we present a novel methodology to integrate multi-modal ocular imaging data, obtained from an Argus II user, producing a model with accurate implant placement, retinal morphology, and whole-eye anatomy. We used finite element analysis to calculate the electric fields generated by retinal stimulation and functionalized the anatomical model with multi-compartment cable models of RGCs to predict retinal activity. We validated the model with diagnostic and perceptual threshold measurements from the same patient.

## II. MATERIALS AND METHODS

### A. Human Subject Imaging

We recruited an eligible participant from the W.K. Kellogg Eye Center (University of Michigan, Ann Arbor, MI). The patient had the Argus II retinal prosthesis implanted in 2015, in the left eye. We obtained informed consent following approval from the University of Michigan’s Institutional Review Board. The study adhered to the tenets of the Declaration of Helsinki and national regulations for medical device clinical trials (NCT03635645).

**Fig. 1.**
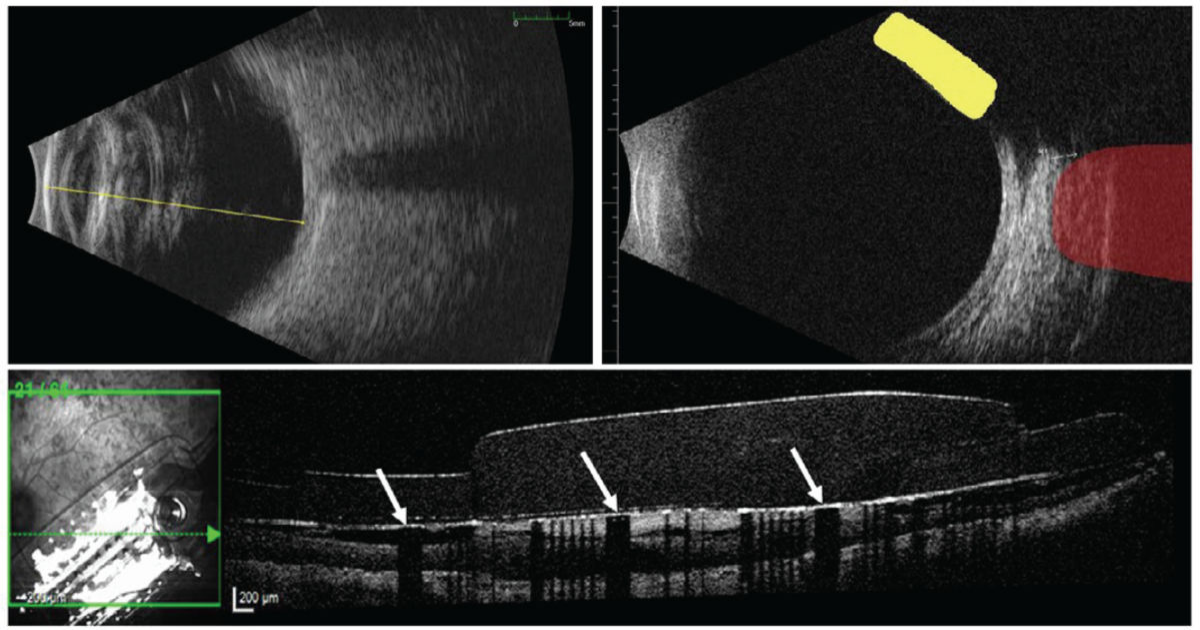
Ultrasound and OCT. The ultrasound on the top left shows axial length (yellow line). The ultrasound on the top right highlights the lateral rectus (red) and Argus EOC (yellow). The OCT scan on the bottom shows the implant, fibrotic tissue, and retinal morphology. Due to its composition, fibrotic tissue appears hyper reflective. Electrodes (white arrows) reflect light from the source, casting dark shadows on the underlying tissue.

Trained technicians obtained ultrasound and optical coherence tomography (OCT) images (Figure 1). We used axial B-scan ultrasound to measure axial length and anterior chamber depth. We used longitudinal and transverse B-scan ultrasound to measure horizontal and vertical vitreous body diameter, along with the angle of the extraocular electronics case (EOC) in the coronal plane. For each dimension, we calculated the average across five images. OCT scans were centered over the MEA, spanning 30° x 25° of the visual field, using 62 sections. Each B-scan was 768 pixels (8.8 mm) by 496 pixels (1.9 mm) and the scan-to-scan spacing was 122 μm. We used OCT for segmentation and reconstruction of retinal morphology.

### B. Experimental Threshold Measurements

**Fig. 2.**
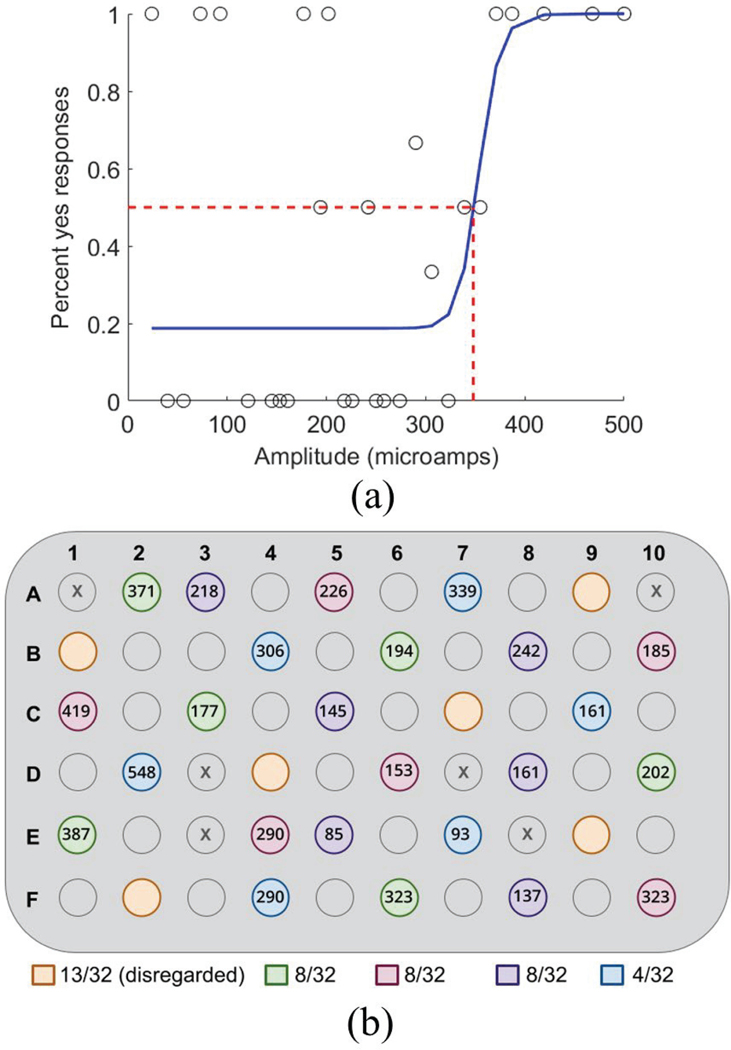
(a) Sample data used to determine the perceptual threshold for electrode A7. Circles indicate patient responses and the blue line is the best-fit sigmoidal curve. (b) The black numbers show perceptual thresholds (μA) for each measured electrode on the Argus II array. Electrode testing blocks are represented by color, and false positive counts are shown below the array for each testing block.

We used a previously established hybrid threshold algorithm to determine the patient’s perceptual threshold for individual electrodes [7]. To mimic stimulation generated by daily Argus II use, all electric stimuli were biphasic, charge-balanced, cathode-first current pulses. The pulse width was 0.45 ms/phase, applied for 250 ms at a pulse frequency of 20 Hz. We generated randomly distributed blocks of six electrodes to test in a series of one-hour sessions. Each session involved 300–400 trials. Each trial administered single-electrode stimulation at a pre-determined pulse amplitude, and the subject responded (verbal “yes”/“no”) based on whether a phosphene appeared. The hybrid algorithm continually generated new pulse amplitudes based on a Weibull distribution of previous responses. We randomized the order of active electrodes and included 32 catch (stimulus-absent) trials per block. Trials continued until the maximum likelihood function converged to 0.5 for each electrode, representing a current amplitude where a phosphene appears 50% of the time (Figure 2(a)). Using 50% percept probability to define visual perception threshold is standard in the field of artificial vision [7], [20], [21]. We tested five blocks, establishing perceptual thresholds for 30 electrodes. However, we disregarded one block due to a high false positive rate (>25% “yes” response for catch trials). Thresholds are shown in Figure 2(b) for the 24 eligible electrodes. Electrode impedance was also measured using the native Argus II hardware.

### C. Patient-Specific Three Dimensional Model

We used parameters from ultrasound to reconstruct the patient’s eye and position the current return (defined as the top part of the EOC) in a three-dimensional (3D) model. We represented the vitreous body as an ellipsoid with x, y, and z diameter determined by ultrasound. We divided the anterior and posterior chambers based on anterior chamber depth. We positioned the EOC tangent to the eye at the equator, and rotated it in the coronal plane according to the angle determined by ultrasound. We estimated remaining anatomical dimensions from literature, including sclera, optic nerve, rectus muscles, and cornea [22]. To represent the head, we included a cylindrical domain surrounding the posterior chamber.

**Fig. 3.**
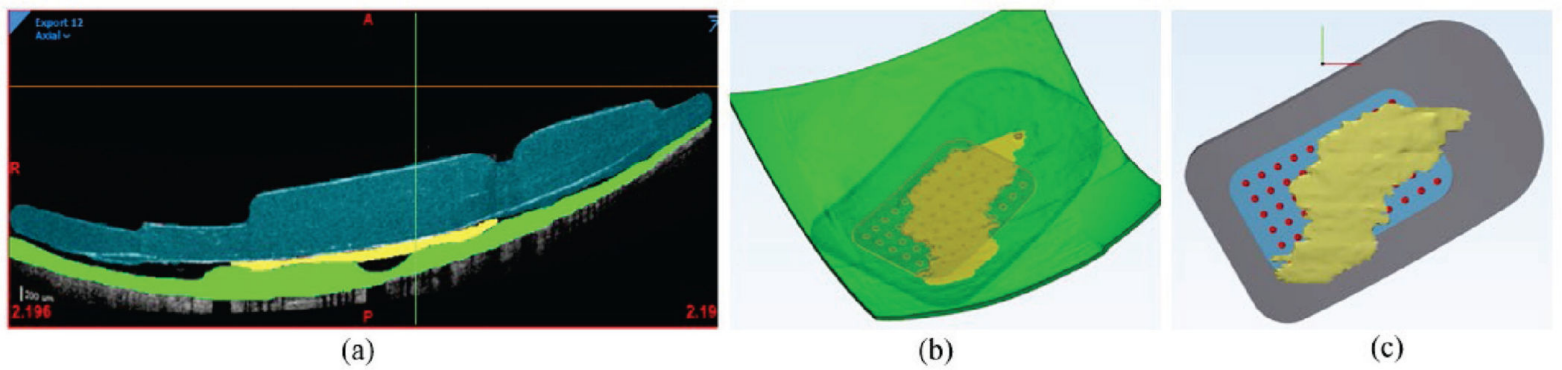
Segmentation and Reconstruction Methods. (a) Segmentation of a single OCT scan. The MEA is shown in blue, fibrotic tissue in yellow, and retina in green. (b) Three-dimensional reconstruction produced by OCT segmentation, isometric view. (c) Implant with fibrotic tissue growth, horizontal view.

We used Mimics Research Version 21.0 (Materialise NV, Leuven, Belgium) to segment OCT scans. Prior to segmentation, we corrected images for posterior shape distortion caused by the OCT display to match the known eye curvature from ultrasound [23]. Although the healthy retina has seven clearly delineated layers, RP causes neuronal migration and degeneration, making it difficult to distinguish between layers [2]. Nystagmus and electrode reflection artifact further limited our OCT resolution. However, previous work indicates that an FEM with seven retinal layers produces similar results to a simplified model including only sclera, retina, and vitreous [17]. Therefore, we represented the retina as a single domain (Figure 3(a)).

**Fig. 4.**
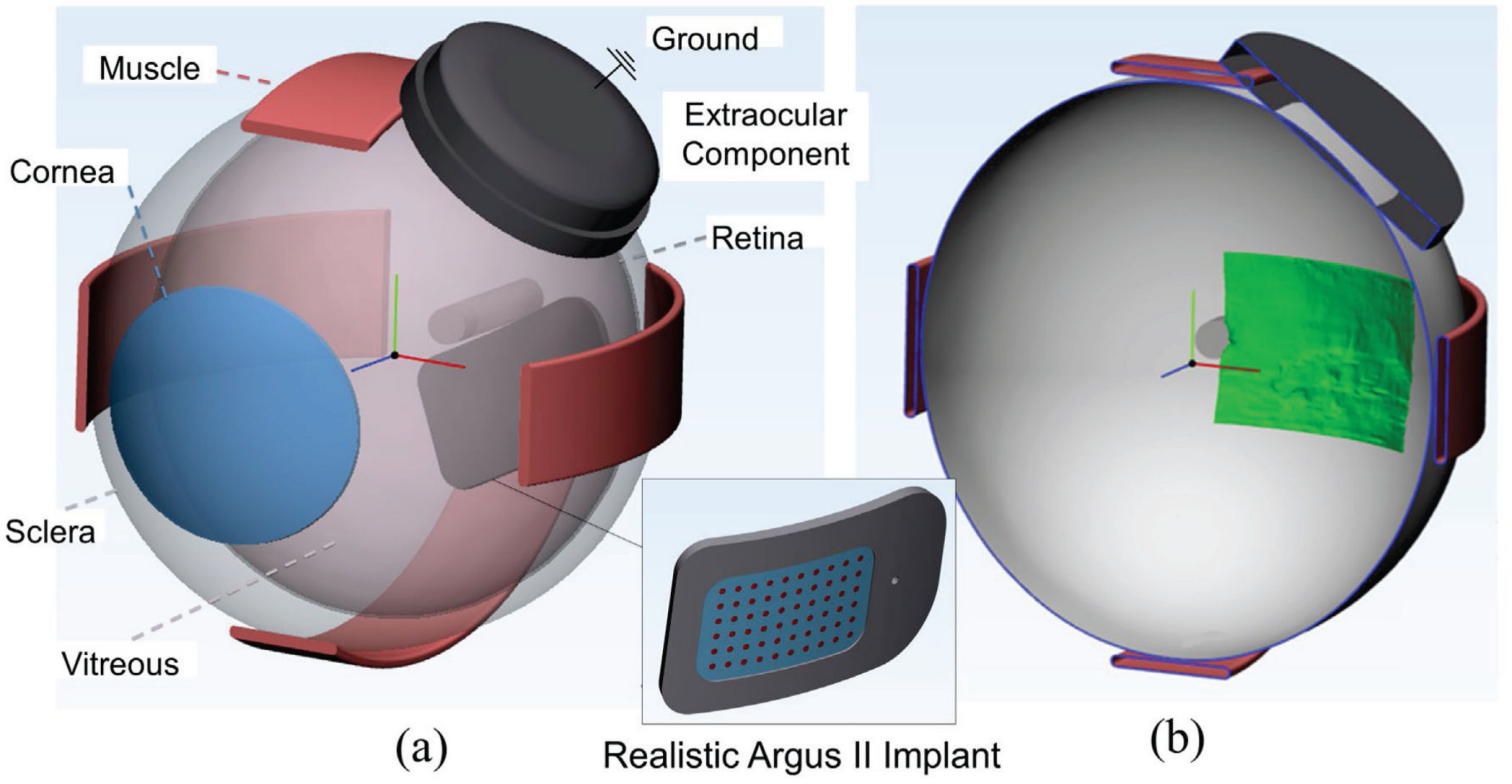
(a) 3D whole-eye model (b) Cross-sectional slice in the horizontal plane showing the position of the segmented retinal mesh from OCT (green).

We used 3-Matic Research Version 13.0 (Materialise NV, Leuven, Belgium) to convert segmented images into a 3D FEM, capturing retinal thickness, MEA position, and fibrotic response (Figure 3(b)). The segmented mesh provided a general outline of the MEA, but lacked component details. Therefore, we used a global distance minimization algorithm to register an accurately detailed Argus II (reproduced with permission from Second Sight Medical Products) with the segmented shape (Figure 3(c)). We then co-registered the segmented surface mesh shown in Figure 3(b) with the whole eye using the optic nerve as a control point. We created a non-manifold assembly to merge all 13 domains, from which we built a volume mesh. We conducted a sensitivity analysis to find the necessary mesh resolution for consistent electric fields and neural thresholds (Table SI). The final patient-specific 3D model is shown in Figure 4.

### D. Simulations

We conducted finite element analysis in COMSOL Multiphysics Version 5.4 (Stockholm, Sweden) using the AC/DC electric currents module. We represented the active electrode as a unit current (I = 1A) terminal, and inactive electrodes as floating potentials. We assigned bulk tissue conductivities (σ) to each domain (Table SII). We used a contact impedance condition to model the thin, resistive retinal pigment epithelium membrane at the boundary between the retina and sclera. We solved the model independently for each electrode. We used a quasi-static solver to calculate electric Potential (ϕ) distribution in our FEM. This solver used the conjugate gradient method to solve the Poisson equation (1).

(1) ∇(σ∇ϕ)=−I

For silicon chronically implanted in the brain, the electrical conductivity of surrounding encapsulation tissue has been measured between 0.15 S/m and 0.37 S/m [24]. We performed a parameter sweep over this range to determine the fibrotic tissue conductivity that produced a model impedance at the center (C5) electrode that best matched with the patient’s average electrode impedance.

**Fig. 5.**
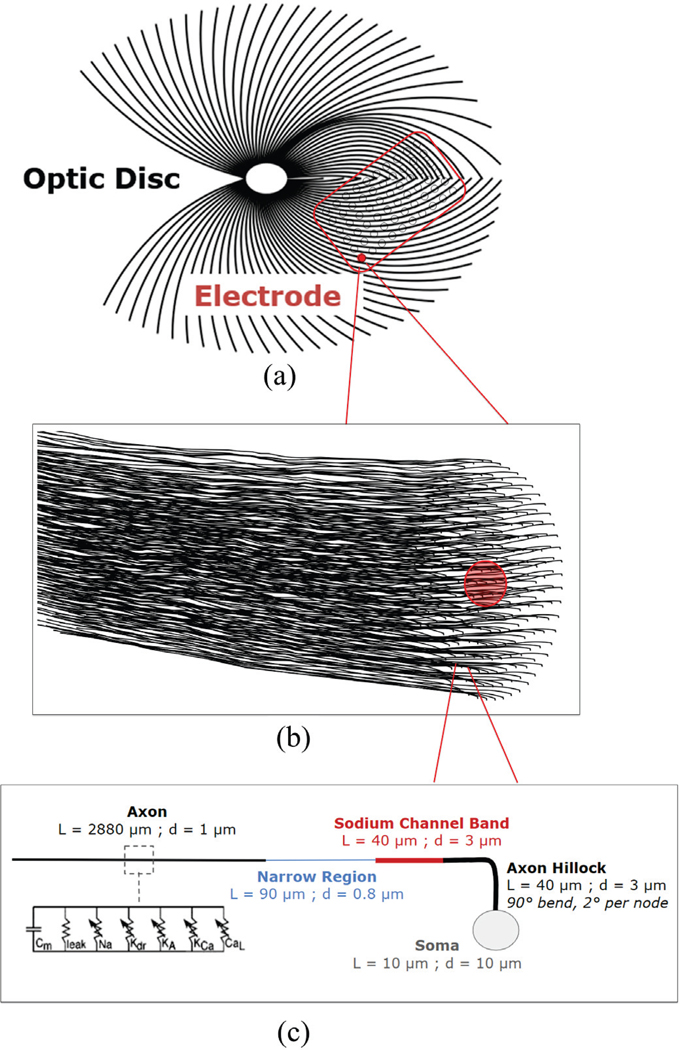
(a) The nerve fiber trajectories [24] with location of electrode array. (b) Sample population of angled neurons (n=250) beneath a single electrode. (c) Schematic of retinal ganglion cell geometry [25] and channel properties [26]. L refers to region length and d to region diameter.

We used biophysical RGC models to predict retinal activity in response to stimulation. For each electrode in our FEM, we uniformly distributed cell bodies within a 500 μm radius of the electrode using Lloyd’s algorithm [25]. Each sample population consisted of 250 neurons and was centered beneath the electrode in the retinal domain [15]. We calculated RGC axon trajectories based on the nerve fiber equations from Jansonius et al (Figure 5(a), 5(b)) [26]. We used this approach to account for the influence of overlying axons on phosphene shape [7]. We positioned each cell body 55 μm below the retinal surface, and axons followed the surface contour of the retina at a 15-μm depth. Following previous work, RGC models had a simplified morphometry with a 90° bend, including a soma, axon hillock, sodium channel band (SOCB), narrow region, and distal axon. (Figure 5(c)) [27], [28].

We used biophysical RGC models to predict retinal activity in response to stimulation. For each electrode in our FEM, we uniformly distributed cell bodies within a 500 μm radius of the electrode using Lloyd’s algorithm [25]. Each sample population consisted of 250 neurons and was centered beneath the electrode in the retinal domain [15]. We calculated RGC axon trajectories based on the nerve fiber equations from Jansonius et al (Figure 5(a), 5(b)) [26]. We used this approach to account for the influence of overlying axons on phosphene shape [7]. We positioned each cell body 55 μm below the retinal surface, and axons followed the surface contour of the retina at a 15-μm depth. Following previous work, RGC models had a simplified morphometry with a 90° bend, including a soma, axon hillock, sodium channel band (SOCB), narrow region, and distal axon. (Figure 5(c)) [27], [28].

We interpolated the spatially dependent FEM solutions to find the extracellular voltage at the center of each compartment in the neuron models. Since biological tissue conductivities are predominantly linear at 20 Hz, we scaled extracellular voltage by the time-dependent stimulus parameters used during experimental threshold measurements (0.45 ms/phase, 20 Hz frequency, 250 msec total). We used previously published Hodgkin-Huxley-type equations to model RGC response to stimulation. The cell membrane included five nonlinear ion channels: sodium $({\bar{g_{\text{N}}}}_{\text{a}}),$ delayed-rectifier potassium $(\bar{g_{k}}),$ A-type potassium $({\bar{g_{k,}}}_{\text{A}}),$ calcium-activated potassium $(\bar{g_{\text{K}}}{,}_{\text{Ca}}),$ and L-type calcium $\left({\bar{g_{\text{C}}}}_{\text{a}}\right)$ [29]. Ion channel conductance varied by region, as described in previous work [28]. We implemented the biophysical cable equations in NEURON v7.7 [30]. Details are provided in the supplementary materials.

We calculated the total RGC length (3,000 μm) required for convergent threshold predictions and set a fixed compartment length of 1 μm to ensure accurate numerical solutions. To calculate the activation threshold for each individual neuron, we used a bisection algorithm to determine the current amplitude (within 0.25 μA) required to induce one action potential per stimulus pulse. We defined visual perception threshold as the minimum stimulus amplitude needed to excite a single neuron for each electrode. Since our model used a reduced neuronal density to estimate retinal activation, single cell activation *in silico* does not necessarily imply single cell activation clinically [15].

## III. RESULTS

We created a patient-specific retinal stimulation model accounting for overall eye shape, EOC position, MEA placement, retinal morphology, and fibrotic tissue growth. We used two co-registered imaging modalities (OCT and ultrasound) to capture these factors for one Argus II patient.

### A. Model Validation

To improve the realism of our patient-specific model, we first set out to match the model electrode impedance to the clinically measured electrode impedances. We calculated impedance for a simulation in which the central electrode (C5) was active, dividing maximum voltage by the stimulus current. In our FEM, a fibrotic tissue conductivity of 0.2715 S/m resulted in an electrode impedance of 8.07 kΩ that closely matched the patient’s average electrode impedance of 8.10 kΩ (7.20–8.90 kΩ).

Next, we compared our model’s RGC activation thresholds with *in vivo* visual perception thresholds. The model predicted an average activation threshold of 402 ± 63 μA (mean ± SD) across all electrodes. The average experimental threshold was 259 ± 116 μA. Previous retinal stimulation models have been developed using cell physiology data, but with highly idealized FEMs of the implant and surrounding tissue. These models report activation thresholds orders of magnitude lower than clinical research studies [14]–[16], [31], [32]. By implementing an existing biophysical model into an anatomically realistic FEM, we predict absolute RGC activation thresholds in an amplitude range similar to perceptual thresholds. We used ANOVA to determine if average perceptual threshold varied between electrode blocks. Results show no significant difference; thus, no substantial threshold drift occurred throughout the day (p-value=0.207).

**Fig. 6.**
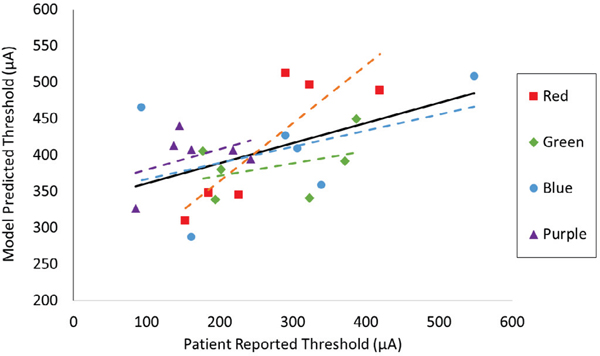
Linear regression analysis comparing *in vivo* and *in silico* threshold data. Each point represents one electrode, and is color-coded based on electrode testing block. A dashed trend line is shown for each testing block, and the solid black line represents the overall trend (r = 0.49)

We also tested correlation of the model’s inter-electrode (n=24) threshold differences with psychophysical inter-electrode threshold differences using least-squares linear regression analysis (Figure 6). We quantified correlation by calculating the Pearson correlation coefficient (r). Overall, model thresholds showed a modest positive correlation with experimental thresholds (r=0.49, p-value=0.014). Removing electrodes with a threshold below 100 μA (n=2) improved the correlation substantially (r=0.56, p-value=0.007). The electrode testing block with the highest average patient-reported thresholds (red) exhibited the strongest correlation with the model (r=0.85, p-value=0.031). We provide rationale for considering these data subsets in the discussion.

### b. Neurophysiological Trends

**Fig. 7.**
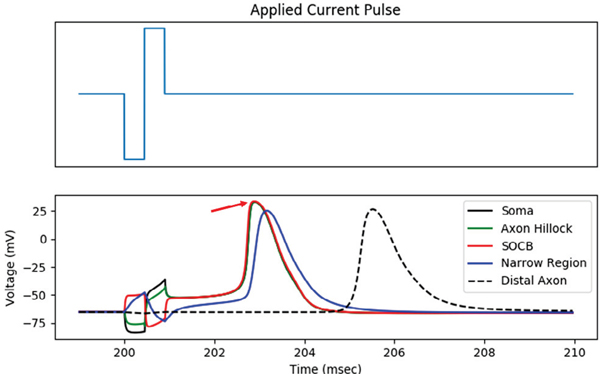
Sample RGC voltage response to an extracellular biphasic current pulse. The action potential initiates in the SOCB, indicated by the arrow.

We defined action potential initiation site as the first neuron compartment in which a spike occurred. At stimulus amplitudes near threshold, we found that the action potential initiation site was consistently located in the SOCB. This trend is corroborated by previous experimental work, which demonstrates that the SOCB is the most responsive site to extracellular electrical stimulation and is the most probable site for spike initiation [33]. Figure 7 shows a simulated RGC response to retinal stimulation. The action potential initiates in the SOCB and exhibits antidromic propagation.

**Fig. 8.**
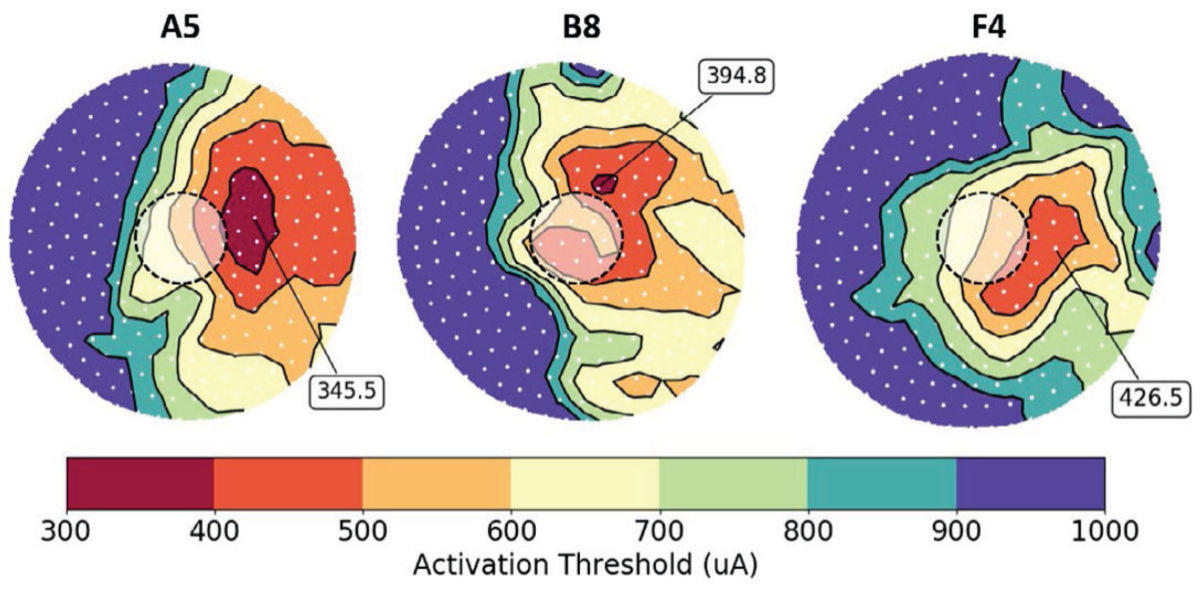
Contour plots showing RGC threshold distribution beneath electrodes A5, B8, and F4. Cell bodies (n=250) are white dots, and electrodes are outlined.

We created contour plots to visualize RGC activation beneath each electrode. These plots show the spatial distribution of cell bodies (white dots), color coded by action potential threshold (Figure 8). Prior experiments have shown that retinal activation is a good predictor for phosphene shape [34]. As such, prior models have used the minimum bounding radius surrounding active neurons as a proxy for phosphene shape [15]. Although it is challenging to quantitatively predict or measure phosphenes, colored activation contours in Figure 8 predict phosphene shape as stimulus amplitude increases.

The model predicts that the lowest current amplitudes do not activate retinal tissue directly beneath the electrode. Instead, the most easily activated tissue is displaced according to the angle of overlying axons. For example, axons beneath electrode A5 have a neutral-slope trajectory (−0.18 < slope < 0.07; axonal slope in the coronal plane, with a sagittal y-axis and transverse x-axis, as depicted in Figure 5(a)). Therefore, electrode A5 activates the SOCB of RGCs that are passing under the electrode, with cell bodies immediately to the right. Axons beneath B8 have a positive-slope trajectory (0.23 < slope < 0.47) and axons beneath F4 have a negative-slope trajectory (−1.46 < slope < −0.59). The contour plots show RGC activation in the positive and negative directions, respectively. This neurophysiological trend is supported by previous work, which has shown that the orientation of phosphene drawings is correlated with axon angle [7].

Among these three electrodes, F4 has the highest visual perception threshold. The electrode is furthest away from the retina, and thus the RGCs are most difficult to activate. Conversely, electrode A5 has the lowest visual perception threshold because it is closest to the retina, and RGCs are more easily activated. Our model correctly predicts these relative threshold differences by capturing variability in electrode-retina distance.

## IV. DISCUSSION

The computational framework presented here improves upon existing models of retinal stimulation by incorporating patient-specific anatomy and electrode locations to quantitatively predict perceptual variability. The model is validated by its ability to reproduce impedance values and threshold amplitudes in a clinically relevant range. Furthermore, threshold predictions *in silico* are positively correlated with *in vivo* perceptual threshold measurements from the same Argus II patient (r = 0.49). A prior study compared electrode-retina distance to perceptual threshold between patients, and found a significant linear correlation (r=0.71, p=0.0002, n=703 electrodes) [8]. However, when we measured electrode-retina distance at the center of each electrode and compared with clinical perceptual thresholds, we found a weak and non-significant correlation (r=0.28, p=0.18, n=24 electrodes). In this case, electrode-retina distance does not offer a complete explanation for inter-electrode perceptual threshold differences, supporting the development of patient-specific models that capture multiple effects in a three-dimensional FEM. Our model is further distinguished from a basic linear model because we derive activation thresholds using first principles of electrical stimulation and cell physiology. As a result, modelled RGC activation follows known neurophysiological trends. This suggests the potential utility of this framework for investigating novel stimulation strategies and electrode designs.

A major challenge was obtaining reliable psychophysical data. The patient’s cumulative false positive rate of 24.5% indicates that even in stimulus-absent trials, they were prone to report phosphene perception. This phenomenon could be caused by fatigue or spontaneous background visual activity, which varies throughout the day. The high false-positive rate introduces substantial error to patient data and may weaken correlation with the model. False positives can skew psychometric functions to the left, resulting in artificially low perception thresholds. For this reason, we included additional analysis for data subsets with high visual perception thresholds. With further validation, the patient-specific model will provide information on activation thresholds that can augment or supersede perceptual threshold measurements for patients with high false positive rates. In the future, we plan to collect phosphene drawings from patients and compare them with model predictions of activation in terms of size, orientation, and elongation [7].

We made several assumptions that could limit our model’s ability to reproduce visual perception phenomena. First, we solved our FEM with isotropic tissue conductivities. However, Esler et al. have proposed that the local uniformity of RGC axon orientation may introduce anisotropy to inner retinal layers [13]. This may affect current flow, altering the extracellular voltage distributions and resultant neural activation. Unfortunately, it is not currently possible to measure retinal tissue anisotropy in patients. Secondly, several studies have reported effects of foveal eccentricity on perceptual threshold, as a result of varying RGC density [8], [31], [35]. Future models should incorporate RGC density. Third, we used an existing block compartment biophysical model which was developed using data from a tiger salamander RGC [28], [29]. Although recent publications have introduced mammalian RGC models, their morphological complexity makes it difficult to systematically place them in the retinal domain of our FEM [36], [37]. In the future, we could augment our framework with a more complex biophysical model that is robust to mammalian temperatures (by incorporating experimentally defined Q10 values) and RGC subtype (by including distinct ON and OFF cells). Finally, we assumed that epiretinal stimulation causes direct RGC activation, disregarding the indirect activation of bipolar and amacrine cells in the retinal network. In the future, we may also incorporate biophysical models of network activation into our patient-specific framework.

## V. CONCLUSION

This feasibility study introduces a novel patient-specific computational framework for retinal stimulation, which we have implemented and validated for one Argus II patient. Using ocular imaging to incorporate critical factors related to retinal morphology and device placement, our model can predict inter-electrode differences in RGC activation. This provides important insight towards retinal activation patterns that cause phosphene variability to occur clinically. In future studies, we will apply this approach to a larger patient cohort with the goal to individualize electrical stimulation paradigms and obtain better retinal prosthesis performance.

## Supplementary Material

supp1-3001563

## Impact Statement—

This proof-of-concept study introduces a novel computational framework that models retinal stimulation on a patient-specific basis to elucidate mechanisms of phosphene variability.
