# Supplementary material for: A Patient-Specific Computational Framework for the Argus II Implant
Source: IEEE Open J Eng Med Biol. 2020 Jun 11;1:190–6. doi: 10.1109/OJEMB.2020.3001563 (PMC7971167; doi:10.1109/OJEMB.2020.3001563)
Supplement: Supplementary materials [file supp1-3001563.pdf]

Supplementary Materials

A Patient-Specific Computational Framework for the Argus II Implant

Kathleen E. Finn, Hans J. Zander, Robert D. Graham, Scott F. Lempka, and James D. Weiland*, *Fellow, IEEE*

Table SI shows the mesh properties for each FEM domain. During triangulation, the edge length of surface elements was constrained to keep geometric error below 1% and the growth rate was set to 25%. The final mesh consisted of 11,565,481 Tet4 elements. Table SII shows the bulk tissue conductivities assigned to each tissue or material.

TABLE SI
SURFACE AND VOLUME MESH PARAMETERS

Domain	Surface Resolution *maximum triangle edge length in mm*	Volume Resolution *maximum tetrahedral edge length in mm*
Platinum<br>Polyimide<br>Silicon<br>Fibrotic Tissue	0.05	0.1
Retina	0.10	1.0
Sclera<br>Cornea<br>Optic Nerve<br>Muscle<br>Vitreous<br>EOC	1.0	1.0
Head Cylinder	2.0	5.0

TABLE SII
ELECTRICAL CONDUCTIVITIES

Material	Conductivity (σ) in S/m	Reference
Platinum	9.46×10^{6}	[38]
Polyimide*	7.00×10^{-16}	
Silicon*	1.00×10^{-12}	
Titanium*	2.60×10^{6}	
Vitreous Humor	1.500	[39]
Sclera	0.503	[39]
Cornea	0.400	[39]
Optic Nerve	0.030	[39]
Muscle	0.350	[39]
Retina	0.0198	[17], [40]
Pigment Epithelium	0.001	[36]
Fibrotic Tissue	0.2715	[24]
Head	0.200	[39]

*Properties used from COMSOL Multiphysics Material Properties Library

.

Table SIII shows the ion densities for the biophysical RGC model in each region, as established by Jeng et al [28].

TABLE SIII
ION CHANNEL CONDUCTANCE BY REGION IN THE RGC MEMBRANE

Region	Soma	Axon Hillock	Sodium Channel Band	Narrow Region	Axon
Length (μm)	10	40	40	90	2880
Diameter (μm)	10	3	3	0.8	1
$\bar{g}_{Na}$ (mS/cm^2)	80	70	350	100	70
$\bar{g}_{K}$ (mS/cm^2)	18	18	18	18	18
$\bar{g}_{A}$ (mS/cm^2)	54	-	54	54	-
$\bar{g}_{Ca}$ (mS/cm^2)	1.5	-	1.5	-	-
$\bar{g}_{K,Ca}$ (mS/cm^2)	0.065	0.065	0.065	0.065	0.065

The equations used to solve for the RGC response to stimulation were derived in the seminal works of Fohlmeister, Coleman, and Miller [29]. The equations are shown below. Code is freely available online, via the Model DB database (https://senselab.med.yale.edu/modeldb/).

The RGC membrane potential in each compartment was modelled by SI. The resting membrane potentials were E_{pas} = -65 mV, E_{Na} = 35 mV, E_K = -75 mV, and E_{Ca} = 132 mV. The axial membrane resistance was 100 Ω-cm and the membrane capacitance (C_m) was 1 μF/cm^2. Due to the paucity of experimentally derived Q10 values, we conducted simulations at 22°C.

$$C_m \frac{dV}{dt} + \bar{g}_{Na} m^3 h (V - V_{Na}) + \bar{g}_{Ca} c^3 (V - V_{Ca}) + (\bar{g}_K n^4 + \bar{g}_A a^3 h_A + g_{KCa})(V - V_K) + \bar{g}_L (V - V_L) = I \quad \text{(SI)}$$

Sodium, calcium, delayed rectifier potassium, and A-type potassium channels are voltage-gated according to state variables. State variables (m, h, c, n, a, h_A) follow the first order kinetic equations of the general form shown in Equation SII.

$$\frac{dx}{dt} = -(\alpha_x + \beta_x)x + \alpha_x \quad \text{(SII)}$$

Specific rate constants for each ion channel are shown in Table SIV. The calcium-dependent potassium channel is ligand-gated according to calcium ion concentration (Equation SIII, SIV).

$$g_{K,Ca} = \bar{g}_{K,Ca} \frac{([Ca^{2+}]_i/(Ca^{2+})_{diss})^2}{1 + ([Ca^{2+}]_i/(Ca^{2+})_{diss})^2} \quad \text{(SIII)}$$

$$\frac{d[Ca^{2+}]_i}{dt} = \frac{-3I_{Ca}}{2Fr} - \frac{([Ca^{2+}]_i - [Ca^{2+}]_{res})}{\tau_{Ca}} \quad \text{(SIV)}$$

TABLE SIV
RATE CONSTANTS FOR VOLTAGE-GATED ION CHANNELS

State Variable	α_x	β_x
m	$\frac{-0.6\,(E+30)}{e^{-0.1\,(E+30)}-1}$	$20\,(e^{-(E+55)/18})$
h	$0.4\,(e^{-(E+50)/20})$	$\frac{6}{1+e^{-0.1\,(E+30)}}$
c	$\frac{-0.3\,(E+13)}{e^{-0.1\,(E+13)}-1}$	$10\,(e^{-(E+38)/18})$
n	$\frac{-0.02\,(E+40)}{e^{-0.1\,(E+40)}-1}$	$0.4\,(e^{-(E+50)/80})$
a	$\frac{-0.006\,(E+90)}{e^{-0.1\,(E+90)}-1}$	$0.6\,(e^{-(E+30)/10})$
h_A	$0.04\,(e^{-(E+70)/20})$	$\frac{0.6}{1+e^{-0.1\,(E+40)}}$
